# Preparation and application of patient-derived xenograft mice model of colorectal cancer

**DOI:** 10.22038/IJBMS.2022.67445.14780

**Published:** 2023-02

**Authors:** Yutao Zhang, Yongming Yang, Likun Zan, Jing Wang, Lei Yan, Lili Zhao, Lixia Chen, Yanfeng Xi, Wenqi Bai, Xihua Yang

**Affiliations:** 1 Department of Colorectal and Anal Surgery, Shanxi Province Cancer Hospital/Shanxi Hospital Affiliated to Cancer Hospital, Chinese Academy of Medical Sciences/Cancer Hospital Affiliated to Shanxi Medical University, 030013, Taiyuan, Shanxi, China; 2 Laboratory Animal Center, Shanxi Province Cancer Hospital/Shanxi Hospital Affiliated to Cancer Hospital, Chinese Academy of Medical Sciences/Cancer Hospital Affiliated to Shanxi Medical University, 030013, Taiyuan, Shanxi, China; 3 Department of Pathology, Shanxi Province Cancer Hospital/Shanxi Hospital Affiliated to Cancer Hospital, Chinese Academy of Medical Sciences/Cancer Hospital Affiliated to Shanxi Medical University, 030013, Taiyuan, Shanxi, China; # These authors contributed eqully to this work

**Keywords:** Alpha-fetoproteins, Cadherins, Capecitabine, Carcinoembryonic antigen, Colorectal neoplasms, Heterografts

## Abstract

**Objective(s):**

Patient-derived xenograft (PDX) model becomes a more and more important tool for tumor research. This study aimed to establish a colorectal cancer PDX model and verify its applicability.

**Materials and Methods:**

Fresh human colorectal cancer tissue was surgically removed and subcutaneously inoculated into immunodeficient mice to establish the PDX model. Hematoxylin and eosin (HE) staining and immunohistochemical staining were used to evaluate the model. The successful PDX model was selected to study the efficacy of capecitabine in treating colorectal cancer.

**Results:**

HE staining showed that the PDX mice model of colorectal cancer could preserve the histological characteristics of the primary tumor. Immunohistochemistry staining showed α-fetoprotein (AFP), carcinoembryonic antigen (CEA), and E-cadherin were strongly positively expressed in primary human and PDX tumor tissues, with a high degree of similarity. Capecitabine significantly inhibited PDX tumor growth and reduced the expression of AFP and CEA proteins in the tumor tissues (all *P***s**<0.05).

**Conclusion:**

We successfully established a colorectal cancer PDX model, and the PDX model could retain the histological and biological characteristics of the primary tumor. Using this PDX model, we revealed that capecitabine at a dose of 300–400 mg/kg can effectively treat colorectal cancer, and no significant difference in toxicity was found among different dose groups. The current work provides a feasible framework for establishing and validating the PDX tumor model to better facilitate the evaluation of drug efficacy and safety.

## Introduction

Colorectal cancer is the third most common malignancy worldwide, with 1.3–1.4 million new cases annually. The occurrence and development of tumors are closely related to the abnormal expression of many genes. E-cadherin is an important epithelial cell marker for epithelial-mesenchymal transformation and plays a key role in the metastasis of tumor cells (1). Carcinoembryonic antigen (CEA) and α-fetoprotein (AFP) are important biomarkers for diagnosis and prognosis of colon cancer (2). At present, the treatment of colorectal cancer mainly involves surgical resection, chemotherapy, and radiotherapy (3, 4). Capecitabine is an oral fluorouracil formulation for the treatment of advanced primary or metastatic colon cancer. After administration, it can be absorbed quickly to exert a fast effect. Capecitabine can be combined with a variety of chemotherapy drugs for cancer treatment (5).

The construction of ideal humanized animal cancer models is very important for the development of new antitumor drugs. The traditional cell line transplantation tumor model is established by inoculating human cell lines into immunodeficient mice, but such models lack the tissue heterogeneity and tumor microenvironment seen in actual human tumors (6, 7). Therefore, when evaluating new antitumor drugs, it cannot truly reflect the efficacy of the drugs in the human body.

In recent years, the patient-derived xenograft (PDX) model has gradually developed into an important tool for tumor research. The PDX model is obtained by transplanting fresh human tumor tissue into immunodeficient mice after processing. Compared with the cell line transplanted tumor model, the PDX model better preserves the human tumor tissue heterogeneity and microenvironment. It also better reproduces the biological process of primary tumor occurrence and development, providing the most clinical-relevant *in vivo* model for tumor research (8, 9). The PDX model also provides a reliable tool for studying the mechanisms of tumorigenesis, drug research and development, and individualized treatment of patients with colorectal cancer (10, 11). This study intended to establish a colorectal cancer PDX model and explore the therapeutic effect of capecitabine for the treatment of colorectal cancer.

## Materials and Methods

***Human colorectal cancer tissue collection***

A 67-year-old female patient (TNM stage of T4bN+M1, IV) underwent colorectal cancer resection but did not receive chemoradiotherapy. Pathological diagnosis of the tumor revealed a differentiated ulcerative adenocarcinoma in the ascending colon, which infiltrated the submucosa of the intestinal wall without the involvement of the nerve tissues. The patient signed an informed consent and agreed to the use of her tumor tissue for this study. This study was approved by the Medical Ethics Committee of Shanxi Provincial Cancer Hospital and the Experimental Animal Welfare Ethics Review Committee. Fresh colorectal cancer tissue (P0 generation tumor tissue) was collected from this patient, placed in Dulbecco’s Modified Eagle Medium/F-12 (DMEM/F-12; Gibco, USA), and cut into 2×2×2 mm pieces on an ice bath.

***Animal preparation and establishment of colorectal cancer PDX model***

Two female NOD/Shi- *scid IL2rγ* ^null^ (NOG) mice aged 7–8 weeks and weighing 20±2 g were purchased from Beijing Vital River Laboratory Animal Technology Co, Ltd. (Beijing, China). The animals were raised in the specific pathogen-free environment of the Experimental Animal Center of Shanxi Provincial Cancer Hospital (License number for experimental animals: SYXK (Jin) 2017-003). After anesthesia of the mice with 2% chloral hydrate (Aladdin) at a dose of 300–400 µl/20 g body weight, the hair was removed at 3–5 mm above both sides of the costal margin (underarm). The area was disinfected with 75% alcohol (Taiyuan Qingxu Kangjiu Pharmaceutical Accessories Welfare Factory, China), and the prepared human tumor tissue (within half an hour after sampling) was subcutaneously inoculated. Then, the PDX model with growing P1 generation tumor cells was established.

Tumor volume was calculated following the formula: V=1/2×l (length)×W(width)^2^, where L is the long diameter, and W is the short diameter of the tumor. When the P1 generation tumor grew to about 1000 mm^3^, the mice were anesthetized with chloral hydrate, and the tumor surface mucous and connective tissues were removed under ice bath conditions. Next, the tumor mass was cut open, and the necrotic part was removed. Then, the processed P1 generation tumor tissue was inoculated into the axilla of 20 female NOG mice aged 7–8 weeks and weighing 20±2 g, according to the above tumor inoculation method, to establish the PDX model with P2 generation tumor subculture for further pharmacodynamics study (12).

***Hematoxylin and eosin (HE) and immunohistochemical staining***

Tumor tissues from patients and PDX mice were fixed with 10% neutral formaldehyde for 48 hr, embedded in conventional paraffin, and sectioned (3–5 μm). Then, the tumor slides were stained with hematoxylin and eosin, dehydrated, rendered transparent, fixed, and sealed. The pathological structure of the tumor tissue was observed under a microscope. The relative expression levels of AFP, CEA, Ki67, and E-cadherin (antibodies from Wuhan Servicebio Technology Co, Ltd., China) in tumor tissues were detected by immunohistochemistry in routine paraffin sections following the standard protocol.

For Ki67 immunostaining, 3–5 visual fields were selected to capture images. The positive staining rate of the nuclei was calculated using the following formula: positive staining rate =number of nuclei with positive staining/ total number of nuclei. For AFP, CEA, and E-cadherin immunostaining, 3–5 visual fields were randomly selected from each tissue section to capture images, and the images were imported into Image PlusPro 6.0 software to calculate the positive area and integrated optical density (IOD) in each image. The average optical density (AOD) was calculated using the following formula: AOD=IOD/area.

***Capecitabine treatment***

The PDX mice with P2 generation tumors were randomly divided into four groups: model, high-, medium-, and low-dose capecitabine groups, with five animals in each group. Capecitabine (Jiangsu Hengrui Pharmaceuticals Co, Ltd., batch number 190921KF) was dissolved in distilled water. Drug administration started when the tumor volume reached 80–100 mm^3^. The model group was given 0.2 ml of normal saline by gavage, and the capecitabine groups were given 200 mg/kg (low-dose), 300 mg/kg (medium-dose), and 400 mg/kg (high-dose) capecitabine, once a day, for 14 consecutive days, and then were stopped drug use for 7 days (13).

***Assessment of tumor inhibition rate***

During the experiment, the survival and conditions of the animals were observed daily. Body weight changes were recorded. Tumor size was measured regularly. If the animal was lethargic, had reduced activity, and the body weight dropped below 18 g, drug administration was stopped, and the mice were noted. Then, according to the situation, 0.1–0.2 ml of 40% glucose could be appropriately given by gavage. The animals were sacrificed by cervical dislocation 100 days later. The tumor mass was dissected, weighed, and photographed, and the tumor inhibition rate of each group was calculated according to the following formulas:

Tumor volume inhibition rate (%) = (V_model _–V_capecitabine_)/V_model_

Tumor weight inhibition rate (%) = (W_model _–W_capecitabine_)/W_model_

Note: V_model_ was the tumor volume in the PDX model group at the end of the experiment, V_capecitabine_ was the tumor volume in the capecitabine group at the end of the experiment, W_model _was the tumor weight in the PDX model group at the end of the experiment, and W_capecitabine_ was the tumor weight in the capecitabine group at the end of the experiment.

***Assessment of organ index***

The liver, kidney, and spleen of the animals were taken and weighed at the end of the animal experiments, and the organ index was calculated as:

Organ index=Organ weight (g) /Body weight (g)×100%

***Laboratory examination***

Blood was collected from each group of mice. A blood cell analyzer (Shenzhen Mindray Bio-medical Electronics Co, LTD. Model: BC-2800VET) was used to determine the blood routine indicators.

***Western blot analysis***

The protein expression levels of AFP and CEA were determined by western blot (WB). RIPA buffer (Beyotime Institute of Biotechnology, Haimen, China) was added to the sample for cell lysis, and protein concentration was determined using the BCA method. A total of 20 μg of proteins was separated by sodium dodecyl sulfate-polyacrylamide gel electrophoresis (SDS-PAGE; Bio-Rad, Hercules, CA, USA) and transferred to polyvinylidene fluoride (PVDF) membrane (Millipore Corp., Billerica, MA, USA). After blocking with 5% bovine serum albumin (BSA) for 1 hr, the membrane was incubated with AFP antibody (mouse, 1:1000; Wuhan Servicebio Technology Co, Ltd., China), CEA antibody (mouse, 1:1000; Wuhan Sanying Biology Technology Co, Ltd., China) and actin antibody (rabbit, 1:2000, Wuhan Servicebio Technology Co, Ltd., China) overnight in a refrigerator at 4 °C. The next day, the membrane was washed with Tris-buffered saline with 0.1% Tween^®^ 20 detergent (TBST) three times, 10 min each time, and incubated with horseradish peroxidase (HRP)-labeled sheep anti-mouse secondary antibody (1:2000) at room temperature for 1.5 hr. The membrane was washed with TBST three times, 10 min each time. A fresh luminescent solution was prepared, and the membrane was incubated for 2 min. Enhanced chemiluminescence (ECL) (Pierce Chemical, Dallas, TX, USA) was used for film exposure and development (Gelview 6000M). The blots were scanned and analyzed using the Image J software (National Institutes of Health, Bethesda, MD, USA).

The average gray value (A) of each strip was analyzed using the Quantity one image analysis system, and the A value of the target strip was divided by the A value of the internal reference actin for normalization. The relative expression level of each detected protein to actin was calculated.

***Statistical methods***

IBM SPSS Statistics 21 was used for statistical analysis, and the results are expressed as mean ± standard deviation. For comparison among groups, one-way analysis of variance (ANOVA) was used for data with a normal distribution. Non-parametric tests were used for data with a non-normal distribution. The comparisons of the two groups were performed using the *t*-test. A *P*-value less than 0.05 was considered to indicate statistically significant.

## Results

***PDX colorectal tumor well reserved the histopathological characteristics of the primary tumor***

**Figure 1:**
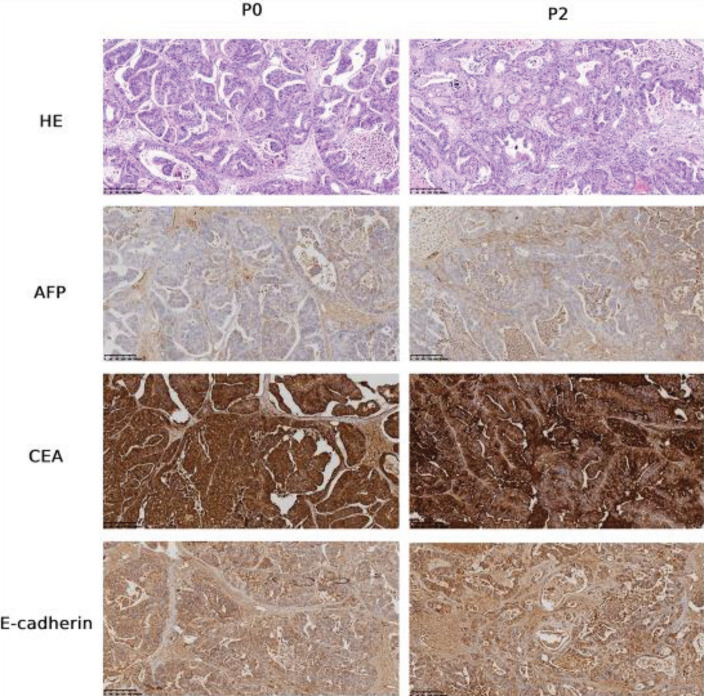
HE staining and immunohistochemical staining of AFP, CEA, and E-cadherin in primary tumor tissue (P0) and P2 generation PDX tumor tissue

We first performed HE staining and immuno-

histochemistry staining to confirm the similarity between PDX colorectal tumor and primary tumor since they were derived from the same source. As shown in the HE staining results in Figure 1, compared with the primary tumor tissue, the transplanted tumor of the P2 generation PDX model showed high structural similarity. In addition, immunohistochemistry staining demonstrated that AFP, CEA, and E-cadherin showed strong positive expressions in both tumor tissues, indicating that the biological characteristics of the primary tumor were well preserved in the colorectal cancer PDX model.

***Capecitabine exhibited therapeutic effects in slowing down tumor growth in the established PDX model***

**Figure 2.**
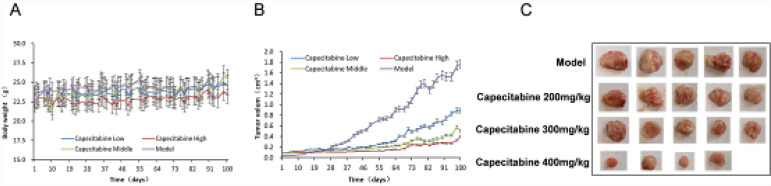
Capecitabine suppressed tumor growth in mice inoculated with P2 generation PDX tumor

We next utilized the P2 generation PDX model to evaluate the therapeutic effects of capecitabine. During the period of animal experiments, all mice in each group had good health condition, and their appearance, feeding, and drinking were normal. The body weight of the mice in the high-dose capecitabine group was slightly lower than that in other groups, but there were no significant differences among the groups (all *Ps*>0.05) (Figure 2A). One mouse in the high-dose capecitabine group died; no abnormal anatomical findings were identified, suggesting that death might be related to the drug’s toxicity. 

The growth rate of the PDX tumors in the model group was the fastest, followed by the low-dose capecitabine group, while tumor growth in the medium- and high-dose capecitabine groups was slower (Figure 2B). At the end of the experiment, the tumor volume in the model group was 1.76±0.23 cm^3^, and those in the low-, medium-, and high-dose groups were 1.01±0.49 cm^3^, 0.57±0.33 cm^3^, and 0.40±0.38 cm^3^, respectively (*P<*0.05 *vs*. the PDX model group). The volume inhibition rates were 42.61%, 67.61%, and 77.27%, respectively (Table 1). After ending the survival observation and subsequent sacrifice of mice, the tumor was dissected and weighed (Figure 2C). The tumor weight of the model group was 2.73±0.49 g, and the tumors in the medium-dose (0.878±0.43 g) and high-dose (0.878±0.43 g) capecitabine groups were significantly smaller (*P<*0.05). The tumor weight inhibition rates in the medium-dose and high-dose groups were 67.77% and 75.09%, respectively (Table 1).

***Capecitabine improved the blood routine indexes of the PDX mice***

The number of leukocytes and the average percentage of monocytes in each group of mice was within the normal range, while the average proportion of lymphocytes was below the normal level, and the average proportion of neutrophils was above the normal range (Table 2). The percentages of neutrophils and monocytes were higher than the reference level, which was consistent with the characteristics of white blood cells (WBC) in immunodeficient animals. The average red blood cells (RBC) and average hemoglobin content were within the normal range, but the average RBC and average hemoglobin values of the model group were lower than those of the normal or capecitabine-treated groups (as shown in Table 2), indicating that capecitabine improved the RBC and hemoglobin levels in the colorectal cancer PDX mice.

***Capecitabine improved organ index in the PDX mice***

Compared with the normal group, the model group had significantly decreased (all *P*s<0.05) liver coefficient, spleen coefficient, and kidney coefficient. Compared with the model group, all capecitabine groups had increased liver and kidney coefficients, and the liver coefficient in the high-dose capecitabine group was significantly increased (all *P*s<0.05). The right kidney coefficient in all capecitabine groups was significantly different from that in the model group (*P<*0.05) (Table 3). Therefore, capecitabine improved the organ index in PDX mice.

***Capecitabine altered the pathological structure and expressions of Ki67 and E-cadherin in the PDX mice***

**Figure 3:**
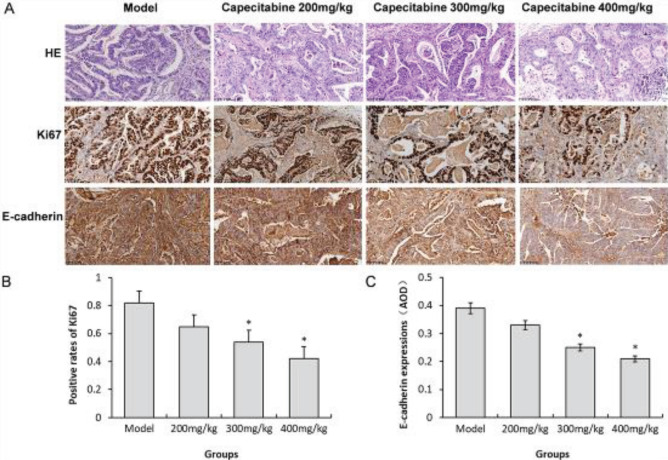
Capecitabine altered the pathological structure and expressions of Ki67 and E-cadherin in tumor tissues of the PDX mice

The model group had more mitotic images and more nuclear atypia, while fewer mitotic images and less nuclear atypia were found after capecitabine administration (Figure 3A). The positive rate of Ki67 staining in the model group was about 80% but decreased to 40%–60% after administrating capecitabine (Figures 3A and 3B). The expression level of E-cadherin was higher in the model group, and the positive signal decreased after the administration of capecitabine (Figures 3A and 3C).

***Capecitabine down-regulated the levels of AFP and CEA in tumor tissues of the PDX mice***

**Figure 4:**
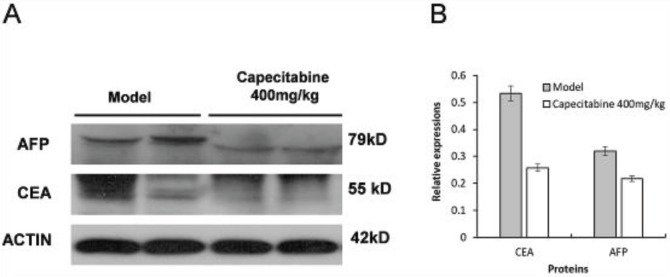
Capecitabine down-regulated AFP and CEA protein expression levels in tumor tissues of the colorectal cancer PDX model

Because high-dose capecitabine had a good antitumor effect, we then compared the expression of AFP and CEA proteins in tumor tissues of the model and high-dose capecitabine groups through western blot assays (Figure 4A). It was found that the expression levels of AFP and CEA in tumor tissues of the model group were higher than those of the treatment groups (Figure 4B). Therefore, high-dose capecitabine intervention could decrease AFP and CEA expressions in tumor tissues.

## Discussion

The PDX model is the preclinical tumor model closest to clinical research so far. It can simulate the histopathology, genome structure, microenvironment, and drug sensitivity of primary tumors. This model simulating human tumor specificity is of great significance for preclinical evaluation, treatment, and tumor prognosis (8, 14, 15). As an oral 5-FU prodrug, capecitabine is increasingly used to treat colorectal cancer (16). Although it is widely regarded as a well-tolerated and low-toxicity therapeutic drug, its cardiac, bone marrow, and gastrointestinal toxicity, hand-foot syndrome, and other aspects cannot be ignored (17-20). This study aimed to explore the application of capecitabine in colorectal cancer and find the appropriate administration concentration. 

The PDX models have been utilized to study the safety and efficacy of many drugs. For example, Maletzki *et al*. (21) explored the relationship between the efficacy, toxicity, and dose of 5-FU by establishing a colorectal cancer PDX model. Yang *et al*. (22) evaluated the efficacy of afatinib in the treatment of HER-2-amplified metastatic colorectal cancer through the PDX model. Jin *et al*. (23) explored the inhibitory effect of capecitabine at 150 mg/kg on tumor formation in colon cancer PDX models. Still, the efficacy and toxicity of different concentrations of capecitabine showed dramatic variations. In this study, the colorectal cancer PDX model was used to evaluate capecitabine’s inhibitory effect and toxicity at concentrations of 200, 300, and 400 mg/kg on colon cancer. 

First, this study evaluated the biological characteristics of tumor tissues of P2 generation PDX mice and primary tumor tissues. Results from HE and immunohistochemical staining demonstrated that the tumor tissue of the colorectal cancer PDX mice retained the morphological and molecular phenotypic characteristics of the patient’s tumor tissue, confirming that the constructed PDX model could represent the original tumor of the patient. These results were consistent with the literature (21). High serum levels of alpha-fetoprotein (AFP) are observed in some gastrointestinal cancers. AFP-producing colon cancers are highly aggressive malignancies with poor prognoses as compared with their non-AFP-producing variant. However, primary AFP-producing colorectal cancer (CRC) is extremely rare(24). The tumor tissue used for the PDX model was from a patient with a highly aggressive disease (TNM stage of T4bN+M1, IV). Since this primary tumor sample is AFP-positive, the unique marker AFP only found in rare cases was supposed to be more specific than other universal colorectal cancer markers in confirming the similarity of pathological characteristics between the primary tumor and tumor from the PDX model.

Several classical markers have been used to recognize colorectal cancer, including carcinoembryonic antigen (CEA), carbohydrate antigen (CA 19.9), tissue polypeptide specific antigen (TPS), and tumor-associated glycoprotein-72 (TAG-72)(25). In addition, recent studies have been conducted on the use of hematopoietic growth factors (HGFs) and various enzymes in the diagnosis and prognosis of colorectal cancer. None of these tests, however, have excellent diagnostic accuracy(25). In our study, besides AFP, we also included one of these well-recognized universal markers, CEA, in our immunochemistry staining. Nevertheless, since most colorectal cancer tissues are non-AFP producing, a combination of the universal colorectal cancer markers is more favorable for confirming the pathological characteristics of PDX tumors. Furthermore, genetic comparisons of the original tumor tissue with the xenograft (as one limitation of this study), such as the approaches of RNA-sequencing and whole-exosome sequencing to identify specific gene mutations, should be more accurate.

The conditions of the animals were generally good during the whole experiment, and only one animal died in the high-dose capecitabine group at the late stage of the experiment. The mice began to decrease food intake and lose weight from 9 days before death and eventually died on the 83rd day of the experiment. Postmortem autopsy revealed no obvious abnormality. This study showed that compared with the model group, high-dose (400 mg/kg) and medium-dose (300 mg/kg) capecitabine could significantly inhibit the growth of colorectal cancer, with a tumor inhibition rate of 75.23% and 67.82%, respectively. The tumor inhibition rate of the low-dose capecitabine group (200 mg/kg) was 35.61%, and no statistically significant difference was seen compared with the model group. Capecitabine had a good inhibitory effect on PDX colorectal tumors, and the effective dose was 300–400 mg/kg. Nevertheless, a previous study showed that 150 mg/kg capecitabine also significantly inhibited tumor formation in colon cancer PDX mice (23). Different primary tumor sources and the number of generations of the original tissue might be the main reasons for the differences.

In addition, this study explored the drug toxicity of capecitabine with different doses in the treatment of colorectal cancer using the PDX model. Blood routine results showed that WBC in all treated mice was within the normal range, which indicated that the overall survival condition of the animals was good. The number of lymphocytes was low, and the number of neutrophils was high, which was consistent with the characteristics of WBC in immunodeficient animals (26). The average RBC and hemoglobin levels were within the normal ranges, but the values of the model group were lower compared with the other groups, indicating that capecitabine could enhance the RBC and hemoglobin levels in colorectal cancer PDX mice. Compared with the model group, the liver coefficient of the drug group was increased to varying degrees and was positively correlated with the tumor inhibition rate. The liver coefficient of the high-dose capecitabine group with the best tumor inhibition effect was the largest and was statistically significant compared with the model group. Therefore, the liver coefficient might be a promising index for prognosis in tumor patients treated with capecitabine.

Furthermore, this study showed that capecitabine treatment significantly reduced Ki-67, AFP, and CEA expression levels in PDX tumor tissues, which was similar to other studies (27). Contrary to previous reports, the results of this study showed that capecitabine reduced the expression of E-cadherin, a marker protein for epithelial cells (28), which may require further experimental analysis.

Representative images are shown. Magnification, ×200

HE: Hematoxylin and eosin; AFP: α-fetoprotein; CEA: Carcinoembryonic antigen; PDX: Patient-derived xenograft

PDX: Patient-derived xenograft

**Table 1:** Tumor weight and tumor inhibition rates of each group of the patient-derived xenograft (PDX) mice

Group	Tumor volume (cm^3^)	Tumor weight (g)	Volume inhibition rate (%)	Tumor weight inhibition rate (%)
Model	1.76±0.23	2.73±0.49	—	—
Capecitabine 200 mg/kg	1.01±0.49*	1.76±0.78	42.61	35.53
Capecitabine 300 mg/kg	0.57±0.33*	0.88±0.43*	67.61	67.77
Capecitabine 400 mg/kg	0.40±0.38*	0.68±0.72*	77.27	75.09

Note: * indicates a significant difference compared with the model group, *P*<0.05

**Table 2:** Blood routine indexes of the patient-derived xenograft (PDX) mice in each group

Blood routine indexes	WBC (10^9^/L)	Lymph (%)	Mon (%)	Gran (%)	RBC (10^12^/L)	HGB (g/L)
Reference range	0.8–6.8	55.8–90.6	1.8–6.0	8.6–38.9	6.36–9.42	110–143
Normal	6.5±1.9	34.6±8.4	5.4±0.7	60.2±6.3	7.66±0.28	123±4
Model	2.4±0.7	32.6±11.1	5.5±1.7	611.±1.0e	6.50±0.95	104±14
Capecitabine 200 mg/kg	3.5±0.6	35.1±16.5	4.9±1.8	60.1±18.2	7.40±0.64	127±11
Capecitabine 300 mg/kg	3.2±0.5	24.3±7.4	6.1±0.6	69.6±8.0	7.09±2.12	117±36
Capecitabine 400 mg/kg	2.6±0.9	27.5±14.6	5.8±1.4	66.7±15.8	7.58±0.15	126±2

WBC: White blood cells; Lymph: Lymphocytes; Mon: Monocytes; Gran: Granulocytes; RBC: Red blood cells; HGB: Hemoglobin

**Table 3:** Organ index of the PDX mice in each group

Group	Liver	Spleen	Kidney	
			Left	Right
Normal	5.08±0.33	0.37±0.10	0.60±0.07	0.61±0.05
Model	3.86±0.35*	0.24±0.16*	0.45±0.01*	0.46±0.01*
Capecitabine 200 mg/kg	4.10±0.12	0.20±0.02	0.48±0.02	0.52±0.05^#^
Capecitabine 300 mg/kg	4.21±0.18	0.23±0.07	0.49±0.02	0.522±0.03^#^
Capecitabine 400 mg/kg	4.26±0.11^#^	0.22±0.04	0.52±0.04^#^	0.54±0.03^#^

Note: *P<0.05 indicates a significant difference compared with the normal group; # *P*<0.05 indicates a significant difference compared with the model group

(A) Representative images of HE staining and immunohistochemical staining of Ki67 and E-cadherin in tumor tissues of the indicated groups. Magnification, ×200. (B-C) The positive rates of Ki67 (B) and E-cadherin expression (C) in the indicated groups were summarized. *P<0.05 indicates a significant difference

compared with the model group

HE: Hematoxylin and eosin; PDX: Patient-derived xenograft

Representative images of western blot bands are shown (A), and the relative expression levels of CEA and AFP were summarized (B). *P<0.05 indicates a significant difference compared with the model group

AFP: α-fetoprotein; CEA: Carcinoembryonic antigen; PDX: Patient-derived xenograft

## Conclusion

We established a PDX model that retained the histological and biological characteristics of the primary tumor. Capecitabine at the dose of 300–400 mg/kg had a significant inhibitory effect on PDX colorectal tumor growth and could improve the RBC and hemoglobin levels in PDX mice. In addition, there was no significant difference in toxicity when comparing medium- and high-dose capecitabine with low-dose capecitabine in this PDX model. The current work provides a feasible framework for establishing and validating the PDX tumor model to better facilitate the evaluation of drug efficacy and safety.

## Authors’ Contributions

YXH, BWQ, and XYF designed the experiments; ZYT, YYM, and WJ performed experiments and collected data; ZLK and YL discussed the results and strategy; ZLL, ZLK, and CLX supervised, directed, and managed the study. All authors approved the final version to be published.

## Funding

This work was supported by the SIGEYIPI Project of the Shanxi Provincial Health Commission (#2020SYS25) and the Scientific Research Project of the Shanxi Provincial Health Commission (#2021076).

## Ethical Statement

This study was approved by the Medical Ethics Committee of Shanxi Cancer Hospital and the Experimental Animal Welfare Ethics Review Committee.

## Conflicts of Interest

The authors have declared that no competing interests exist.
